# A Novel Phage Protein Mediates the Virus's Removal from Bacterial Chromosomes

**DOI:** 10.1371/journal.pbio.0040213

**Published:** 2006-05-30

**Authors:** Liza Gross

Long before anyone realistically entertained the notion of engineering transgenic corn, cloned dogs, or designer babies, scientists looked for clues to the fundamental properties of life in much simpler organisms. Studies in one such organism—bacteria-infecting viruses called bacteriophages, or phages—produced some of the most important discoveries of molecular biology: identifying DNA as the genetic material, elucidating the mechanism of genome replication, and revealing the power of recombinant DNA, the backbone of genetic engineering.

Nearly 60 years after phage's golden era, scientists are still using the virus to illuminate the inner workings of the cell. And now in a new study, Pallavi Ghosh, Laura Wasil, and Graham Hatfull describe a novel phage protein that uses an unusual mechanism to induce the infectious stage of the phage life cycle.

Lacking the means of self-replication, phages inject their genome into a bacterial cell and co-opt its molecular machinery to mass produce new viruses, ultimately destroying the cell to release its progeny. Temperate phages can reproduce through this lytic life cycle or enter a lysogenic life cycle during which they integrate their DNA into the bacteria's genome as a noninfectious “prophage.” The prophage remains harmless until it is excised from the bacterial genome.

Both integration and excision depend on the combined efforts of integrases and recombination directionality factors (RDFs) in a cut-and-connect operation called site-specific recombination. During integration, integrases mediate DNA-strand exchange between phage DNA—at the phage attachment site, *attP*—and bacterial DNA—at the bacterial attachment site, *attB*. Successful integration creates junction sites— *attL* and *attR*—on both sides of the integrated prophage that mark future excision sites. RDFs can catalyze integration or excision, typically by binding to DNA near the respective recombination sites and forming a complex with integrase that remodels the DNA and creates macromolecular structures that promote one process while inhibiting the other.

While much is understood about how tyrosine integrases (so-called because the amino acid tyrosine mediates the catalytic reaction) effect site-specific recombination, Ghosh et al. were interested in the more cryptic serine integrases. Serine integrases lack many of the features other enzymes use to regulate site-specific recombination, such as DNA-binding cofactors or DNA remodeling, and little is known about the few RDFs that have been linked to them. To learn more about serine integrase activity, the researchers worked with a phage called Bxb1 that uses a serine integrase to insinuate itself into the genome of the bacterium *Mycobacterium smegmatis*.

They first analyzed the Bxb1 genome to identify candidate RDFs using standard bioinformatic methods, but RDFs are too diverse to allow accurate sequence comparisons. So they turned to an experimental method to isolate the RDF, using a phage strain with genes organized akin to an integrated prophage, complete with recombination junctions *(attL* and *attR)* and integrase *(int)*. From this strain, they engineered an “excision tester strain” with one stretch of sequence conferring antibiotic resistance and another conferring sucrose sensitivity (thus, stunting growth) inserted between the *attL* and *attR* junctions. Successful excisive recombination would remove the intervening sequence and confer sucrose resistance, allowing the strain to grow in sucrose.

To identify excision-related sequences, Ghosh et al. created a Bxb1 DNA library and incorporated it into the tester strain, producing colonies of genetically transformed strains. The resulting colonies produced sucrose-resistant strains, which had undergone site-specific excision recombination between *attL* and *attR*, the researchers confirmed, which had been mediated by a fragment of Bxb1 DNA and a putative RDF. And by creating genetic variants of the tester strains, they showed that excisive recombination requires both the Bxb1 integrase *gpInt* and a candidate Bxb1 RDF.

Only one region in this putative RDF, gp47, had been previously described. And though it is much larger and unlike any other known RDF, the researchers show that gp47 does indeed catalyze excisive recombination and can inhibit integration, like other RDFs. Bxb1 gp47 distinguishes itself, however, by regulating site-specific recombination through novel interactive mechanisms that don't require complex macromolecular structures or direct DNA binding. Instead, the researchers propose, it instigates excisive recombination through transient interactions with gpInt– *attL/R* junctions—and blocks integrative recombination through more stable interactions that prevent gpInt– *attB* and gtInt– *attP* complexes from forming productive associations.

Altogether, these results point to a novel RDF with an unusual strategy for regulating integration and excision. Though the gp47 RDF acts through protein–protein interactions rather than through direct DNA-binding interactions, its effects depend on the specific DNA site that gpInt binds. With evidence that gp47 also plays a role in DNA replication, the researchers plan to investigate whether this novel enzyme uses the same strategy to interact with other phage proteins—and maybe even to commandeer host machinery and unleash infection.

## 

**Figure:**
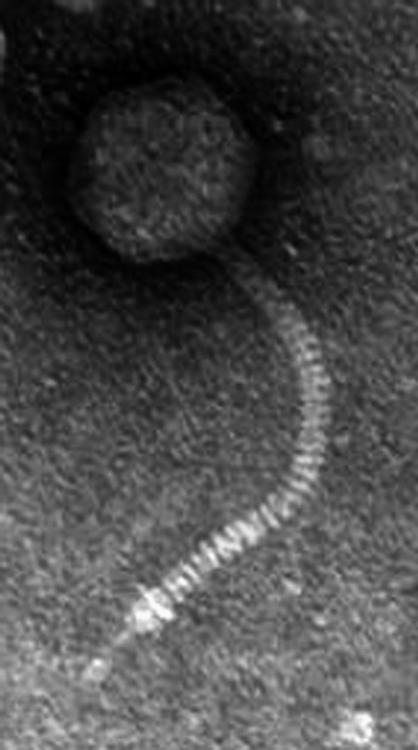
The mycobacteriophage Bxb1 uses a novel enzyme to remove itself from its bacterial host's genome before launching an infection.

